# The current knowledge on centipedes (Chilopoda) in Slovenia: faunistic and ecological records from a national database

**DOI:** 10.3897/zookeys.510.8672

**Published:** 2015-06-30

**Authors:** Blanka Ravnjak, Ivan Kos

**Affiliations:** 1 University of Ljubljana, Biotechnical faculty, Department of Biology, Večna pot 111, 1000 Ljubljana, Slovenia

**Keywords:** Centipede fauna, biodiversity maps

## Abstract

In spite of Slovenia’s very high biodiversity, it has only a few animal groups that have been significantly investigated and are well known in this area. Slovenian researchers have studied only about half of the species known to be living in the country (Mršić 1997), but among well investigated species are centipedes. All available data about centipedes in Slovenia collected from 1921 to 2014 have been consolidated and constitute a general electronic database called “CHILOBIO”, which was created to provide an easy overview of the Slovenian centipede fauna and to allow entry and interpretation of new data collected in future research. The level of investigation has been studied with this database, in conjunction with a geographic information system (GIS). In the study period, 109 species were identified from 350 localities in 109 of the 236 UTM 10 × 10 km quadrants which cover the study area. The south-central part of the country has been the subject of the best investigations, whereas there is an absence of data from the south-eastern, eastern and north-eastern regions The highest number of species (52) has been recorded near the Iška valley (Central Slovenia, quadrant VL68). In 48% of the UTM quadrants investigated fewer than 10 species were recorded and just 5 species were found in one locality. Seventeen species were reported only in the Dinaric region, 4 in the Prealpine-subpannonian region and 7 in the Primorska-submediterranean region.

## Introduction

Centipede research has a very long tradition in Slovenia. In the 18^th^ century the Italian naturalist Giovanni Antonio Scopoli gathered specimens in the surroundings of the mining town of Idrija. Some of these specimens were subsequently studied by C. L. Koch (Eason 1970). Therefore, records concerning centipedes in the Slovenian area have been collected for more than 200 years (Valvasor 2004). Systematic and particularly intensive research has been performed during the last thirty years. In this period, new findings on Lithobiomorpha has been published (Kos 1988). The knowledge on Geophilomorpha also increased (Lesar 2002), especially on *Stenotaenia* C. L. Koch, 1847 (Huzimec 2009) and *Strigamia* Gray, 1843 (Schoss 1991). In addition, the centipede diversity of some areas in Slovenia has been studied in detail (e.g. Kos 1988, 1995, Kos and Praprotnik 2000, Grgič and Kos 2003, Ravnjak 2006, Pagon 2006, Kohek 2012, Bagola 1997). The data gathered allows the conclusion that the centipede fauna in Slovenia is very rich. By 2011, the presence of 98 species, out of 538 in Europe (Engoff 2013), was confirmed in Slovenia. Among the 98, 35 species are endemics to the study area (Kos 2001), and 41.8% are evaluated in the Red List of Endangered Species in Slovenia (Official Gazette of RS No. 82/2002).

The reasons underlying such species diversity include the country’s biogeographic features, as well as paleogographic and paleoclimatic events. A factor that most probably enabled species diversity, is the tectonic evolution of Slovenia, which influenced geology, relief features and edaphic conditions of the country (Mršić 1997). Additionally, climate, resulting from geographical position, and microclimate factors should be noted (Mršić 1997). Slovenia is one of the southernmost European countries. With an area of 20,256 km^2^, it lies at the junction of four geographical macroregions: the Alps, the Pannonian basin, the Dinarides and the Mediterranean. Besides its biogeographic articulation, three climate types interweave: Mountain, Mediterranean and Continental (Ogrin 2004). The annual amount of precipitation decreases from west to the east, and the maximum precipitation in a year can be as much as 3.500 mm. Temperature extremes are also typical, with winter values falling to -30 °C and summer values as high as +40 °C. A total of 514 habitat types have been described in the area and the dominant ecosystem consists of forests, which cover some 56% of the territory (Jogan et al. 2004). Mixed forests predominate and the most important habitat types are colinian and submountainean beech forests, and acidophilic and fir beech forests (Agencija Republike Slovenije za okolje 2001).

Despite various investigations in different areas and habitat types have been carried out, no comprehensive review on which parts are well investigated, from which areas data are still lacking and what the quantity and quality of data about centipedes is in Slovenia have been done. Also no clear overview about the state of centipede research in individual habitat types was available. In this report, all available data concerning centipedes have been collected and maps allowing evaluation of the state of research on centipede fauna in Slovenia are presented.

## Methods

The base for research presented here was the data about centipede findings in the whole area of Slovenia. This data consist from centipede findings between the years 1921 and 2014. In terms of geographical distribution the findings were associated with the 10 × 10 km UTM grid, where the area of Slovenia then is divided into 263 quadrants. The research included all the quadrants also those which only partial cover the Slovenian area. Then a “CHILOBIO” database, in form of a MS Excel table, has been created. The number of species and specimens found in a single UTM quadrant was used as a basis for mapping the state of research on centipedes. The data in this database result from random findings as well as from targeted research. All the specimens gathered within this research are stored in the collection of microscopic and ethanol-preserved preparates at the Department of Biology at Biotechnical Faculty of the University of Ljubljana. Each data record contains the name of the species from a certain finding location along with the number of specimens found and all the accompanying data. Some of the species found are even new for centipede fauna and their descriptions are not published yet. A basic database entry provides species, inventory number of the specimen from this species and the location of the finding. Besides the basic data about each species the database also contains information (elevation above s.l., nearby town) about localities in which species were encountered along with some basic environmental data, date of the collection, data about the collector and the sampling methods. Detailed data was incomplete or missing in most of the random centipede findings, while target research included abovmentioned data. Among the location data, the UTM quadrant where the species was found is included. Subsequently, these quadrants were used as basic cartographic units for representation of the state of research on centipedes in Slovenia. For the majority of the older data, besides the collecting site, only the respective UTM quadrant is used to define the geolocation. The vector layer of the UTM grid was made with the geographical information system software tools (ArcGIS ver. 9.2, ESRI). The state of research on centipedes was represented by the number of species and specimens found within each individual quadrant. The result is a map of Slovenia in which the number of species and specimens per quadrant is presented.

For the evaluation of the state of research within each habitat types, we compared the number of specimens and species recorded. Only that kind of data where the habitat type of finding site was also recorded, where included in this comparison. Such data were mostly collected by quantitative sampling methods, where the area of individual sampling unit and the number of units were known.

## Results

The total number of records stored specimens recorded in the “CHILOBIO” database is 14.835. Records refers to 25,651 specimens describing 109 species. One percent of specimens were not identified to the species level, due to damages of morphological features.

**Figure 1.**
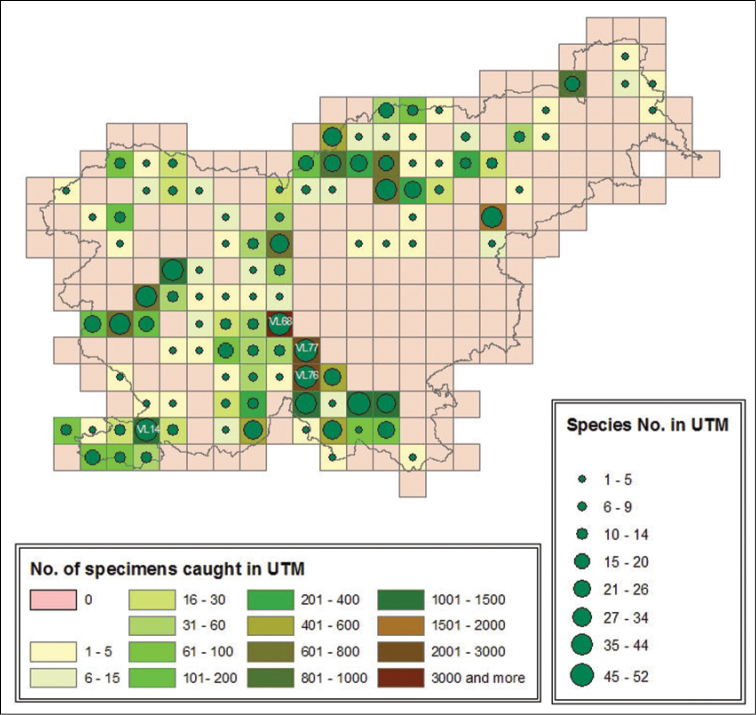
Number of centipede species and specimens caught in individual UTM quadrants from 1921 till 2014 in Slovenia.

To date, species have been found in 350 localities within 105 UTM quadrants, representing 41% of all quadrants in the Slovenian UTM grid. The highest number of species (52) was found in in the quadrant VL68 that lies to the south of the capital, Ljubljana. In terms of the number of species identified, this quadrant is followed by its neighbouring quadrants (VL76 and VL77) with 48 and 43 species found, respectively. In the area south of Ljubljana (including 12 UTM quadrants), which consists mostly of dense Dinaric forests, the number of quadrants with more than 20 species is highest. In terms of number of centipede species found per quadrant one area in western Slovenia (Trnovski gozd area) and one in northern Slovenia (Mountain chain in Upper Savinjska valley along Austrian border) are prominent. In 48% of 105 investigated UTM quadrants, the recorded number of species was fewer than 10.

In terms of number of specimens identified, the quadrant VL68, with 6.479 recorded specimens, leads again. A high number of specimens, but less than 50% of the total, were found in quadrants VL76 (2.264), VL75 (1.479) and VL77 (1.312). These quadrants mostly are those with higher number of species found. All of this four are in the Dinaric geographic region.

Comparing the number of specimens caught in different habitats, the majority (63%) were found in beech forests. Just 8% were found in mixed forests, while 5% and less were found in other habitats. The highest number of species (79) was found in beech forests, while 48 were caught in mixed and 44 in thermophilic forests (Table 1).

**Table 1.** Number of species and specimens recorded per habitat types.

Habitat type	No. of species	No. of specimens
Beech forest	79	9582
Mixed forest	48	1298
Oak forest	44	799
Thermophilic forest	39	563
Grassland	37	374
Frost hollow forest	32	563
Coniferous forest	32	290
Overgrowing meadow	23	214
Hornbeam forest	22	126
Bush	21	325
Dwarf pine stands	19	339
Meadow	19	87
Frost hollow spruce stands	16	337
Floodplain forest	13	40
Alder stands	11	60
Juniper stands	9	30
Anthropogenic habitats	3	333

## Discussion

With such a volume of data there emerged a need for a universal electronic database in which all researchers could store and access the gathered data as necessary. The “CHILOBIO” database will be a source for faunistic and systemized research of various researchers, who will be able to perform different spatial analyses even without additional sampling. Till now this database is placed at the Department of Biology of Biotechnical faculty of Ljubljana and there is not yet a free access to data for anyone, except for the researchers of this faculty. The “CHILOBIO” database is a useful source for spatial research of centipede fauna because it contains some basic parameters, including collecting site coordinates, altitude above sea level, UTM quadrant, habitat type, of each individual specimen and species. By using the basic tools from the geographical information system (GIS) it is possible to create maps of distribution of individual species that support planning of further research (Wadsworth and Treweek 1999). Distribution maps of this sort had been created previously by Simaiakis and Mylonas (2006) for 14 centipede species in the Aegean archipelago. For Slovenia, no such cartographic representation of centipede biodiversity has been made to date and the map presented here is the first of its kind. In this representation the state of research in the study area can easily be seen. However, the high number of registered species and specimens found in certain quadrants is primarily a consequence of sampling effort of targeted research and investigations. Most of this research was performed in the area of Dinarides between Ljubljana, Velike Lašče and Kočevje (Kos 1988, 1995, Grgič 2005, Kohek 2012). Other research was carried out in western Slovenia (Pagon 2006), eastern Slovenia (Ravnjak 2006), Carinthia (Kos et al 2000), NE part of Slovenia (Bagola 1997) or in the Primorska region as far as Snežnik (Kos 2010). Based on this, it can be concluded that low number of specimens and species recorded, does not represent the actual state of art but only the low sampling effort to date invested. Continued research is needed in the future, especially in the SE and NE parts of Slovenia, to establish a comprehensive evaluation of species diversity of centipede fauna. These two areas represent the major part of the Pannonian biogeographical region where findings of species with Pannonian distribution are expected and where knowledge about the distribution of species can be improved. It would be reasonable to supplement the existing cartographic representation with the representation for some species, as was done by Tuf and Laška (2005) for the Czech Republic and by Barber and Key (1988) for the British Isles. The same was done also by Zapparoli and Minelli (2007) for Italy and by Simaiakis et al (2013) for Cyprus. For Slovenia, investigation of the areas along the borders with neighbouring countries is also reasonable as species already found in those countries can be expected there.

Although only about a half of Slovenia has been investigated, that 109 species have already been recorded confirms that centipede fauna of this area is very rich. According to the European Atlas of Soil Biodiversity for the millipede group (Diplopoda Myriapoda), Slovenia is at the top (Jeffrey et al. 2010). These authors estimate that the number of millipede species in Slovenia is expected to be between 151 and 622. Among recorded species, eight await scientific description. Based on the available data (Kos 1992, 1988) the majority of centipede species found in Slovenia (52 species, 48%) have an in-Dinaric distribution, according to biogeographical distribution models proposed by Matvejev and Puncer (1989), while somewhat fewer have ex-Dinaric distribution (41 species, 37%). In addition, only one species has a Pannonian distribution. With respect to general distribution, the majority of species found in Slovenia (19) have a Palearctic distribution, while seven have a mid-European and six a European distribution. For two species the distribution is South-European and for four Mediterranean (Kos 1992). Due to geographical position of Slovenia, the presence of such distribution patterns is expected. Among all species found, 17 were endemic for the Dinaric region, seven for the Mediterranean and four for the Prealpine-subpannonian regions. Several species, including *Lithobius illyricus* Latzel, 1880, *Lithobius peregrinus* Latzel, 1880, *Cryptops umbricus* Verhoeff, 1931, *Geophilus promontorii* Verhoeff, 1898, and a still unidentified *Brachygeophilus* sp., were found only at one site.

Comparing the proportions of centipede specimens found in individual habitat types, we established that beech forests are the most investigated habitats. In part, this can be explained by the fact that research often was targeted at investigations of beech forest fauna (Grgič 2005), but it should be noted that several entries in “CHILOBIO” lack information about habitat type, and the representation of centipede community in individual habitat type is therefore deficient. In future, it would be reasonable to perform sampling in habitat types such as *Acer* forests, pine forests, ash forests, and alder forests where, so far, little research has been done.

## Conclusions

Within this research a general electronic database ‘CHILOBIO’ with data about centipede findings from 1921 until 2014 in Slovenia was made. The data included in this database are a result of random findings as well as from targeted research. This database represents the first collection of data about centipede fauna in Slovenia and also a basis for a cartographic representation of state of research of centipede fauna in this area. Because one of the main data included in database is also a locality, where centipede species were found, based on data in ‘CHILOBIO’, the map with state of research presentation was created. A base for evaluation of well and poorly investigated areas in Slovenia was the number of species and specimens found in 10 × 10 km UTM quadrant. Based on the UTM grid with centipede species and specimens per UTM quadrant, we established that more than one-half of the country is still poorly investigated. Whereas especially Pannonian and Alpine regions are poorly investigated, the area of Dinarides between Ljubljana, Velike Lašče and Kočevje is well investigated. Till now all-together 109 centipede species were found in Slovenia. Some among them are new for centipede fauna and their descriptions are not published yet. As well the database as the cartographic representation can be used for designing future targeted investigations, creation of distribution models for individual species and processing of data gathered during sampling and stored in the database.
